# Synergistic predictive value of MRI-based vertebral bone quality and CT hounsfield units for postoperative sagittal balance deterioration after cervical laminoplasty

**DOI:** 10.3389/fsurg.2025.1722804

**Published:** 2025-12-18

**Authors:** Bin Zheng, Panfeng Yu, Zhenqi Zhu, Yan Liang, Haiying Liu

**Affiliations:** Spine Surgery, Peking University People’s Hospital, Beijing, China

**Keywords:** bone mineral density, cervical laminoplasty, hounsfield unit (HU), sagittal balance deterioration, vertebral bone quality (VBQ)

## Abstract

**Background:**

Sagittal balance deterioration is major concern after cervical laminoplasty, which affects neurological recovery and quality of life. Preoperative bone quality may play a key role. CT-derived Hounsfield Unit (HU) values reflect bone mineral density, while MRI-derived vertebral bone quality (VBQ) values indicate bone marrow fat content. Their combined predictive value remains unclear.

**Methods:**

This study retrospectively analyzes 104 patients (mean age 58.4 ± 11.6 years) who undergo posterior single-door laminoplasty with preoperative cervical CT and MRI. Mean HU and VBQ values (C2–T1) are measured, and patients are classified by postoperative change in cervical sagittal vertical axis (ΔcSVA). Logistic regression and ROC analyses are used to evaluate predictive performance.

**Results:**

HU and VBQ differ significantly among improvement, stable, and deterioration groups (*P* < 0.001). Both HU (−0.007, *P* = 0.009) and VBQ (2.521, *P* = 0.018) independently predict postoperative imbalance. VBQ correlates negatively with HU (*r* = −0.306, *P* = 0.0016). ROC analysis shows AUCs of 0.690 for HU, 0.747 for VBQ, and 0.791 for the combined VBQ + HU model, with improved sensitivity (0.838) and specificity (0.597).

**Conclusion:**

Decreased HU and elevated VBQ independently predict postoperative sagittal balance deterioration, reflecting complementary aspects of vertebral bone quality. The combined MRI- and CT-based assessment improves prediction accuracy and assists in preoperative risk stratification and individualized surgical planning.

## Introduction

Maintaining normal spinal sagittal balance is crucial for postoperative functional recovery and quality of life in patients. Although cervical laminoplasty decompresses the spinal cord by expanding the spinal canal while preserving motion segments, some patients develop cervical kyphotic deformity or loss of physiological curvature during postoperative follow-up, indicating sagittal balance deterioration. This condition may lead to poor neurological recovery or recurrent clinical symptoms (1, 2). Currently, preoperative identification of such high-risk patients represents one of the clinical priorities. Osteoporosis is considered a potential factor affecting postoperative spinal alignment stability. Osteoporosis weakens vertebral mechanical stability and increases the risk of implant subsidence, vertebral collapse, or alignment loss (3–6). Therefore, accurate assessment of preoperative vertebral bone mineral density and bone quality holds important clinical significance for predicting postoperative sagittal balance changes.

Dual-energy x-ray absorptiometry (DEXA)-measured bone mineral density T-scores serve as the gold standard for osteoporosis diagnosis. However, their application faces limitations in patients with spinal degenerative diseases. Degenerative changes, osteophytes, and vertebral sclerosis can cause falsely elevated DEXA results, failing to truly reflect cancellous bone strength within vertebrae. In contrast, clinical practice gradually adopts “opportunistic indicators” from imaging examinations to assess vertebral bone quality, including CT scan-derived vertebral cancellous bone Hounsfield unit (HU) values and MRI T1-weighted image-calculated vertebral bone quality (VBQ) scores. CT-HU values correlate highly with quantitative CT-measured bone mineral density and can be obtained through region of interest (ROI) measurements on routine diagnostic CT scans, reflecting local cancellous bone density while avoiding the influence of degenerative factors (7, 8). MRI-derived VBQ scores reflect bone strength through signal intensity from bone marrow fat components within vertebrae, specifically calculated as the ratio of vertebral cancellous bone signal to adjacent cerebrospinal fluid signal on T1-weighted images (9). Studies show that VBQ values correlate negatively with bone mineral density and can effectively assist in diagnosing spinal osteoporosis (10, 11).

Existing literature reports that for cervical surgery, recent studies indicate that elevated preoperative cervical VBQ scores represent an independent risk factor for distal junctional kyphosis (DJK) following posterior long-segment fusion (12). Similarly, decreased preoperative mean HU values are found to correlate significantly with cervical curvature loss after cervical laminoplasty (13). Single imaging bone quality indicators have demonstrated certain predictive value, but each has limitations. HU values primarily quantify bone mineral density without reflecting trabecular bone quality (14), whereas VBQ qualitatively assesses bone marrow fat content (10, 15).

This study examines patients undergoing cervical laminoplasty, comparing preoperative VBQ and HU value differences between those maintaining good postoperative sagittal balance and those experiencing balance deterioration. The study further establishes a combined VBQ + HU prediction model and evaluates its performance.

## Methods

### Patient inclusion criteria

This study is a retrospective analysis that consecutively includes patients diagnosed with cervical spondylotic myelopathy who undergo posterior single-door laminoplasty at our institution between January 2015 and December 2022. All surgery are performed by one surgeon with 10 year experience. All patients wear a rigid cervical collar postoperatively and undergo a standardized rehabilitation program delivered by the same team. The inclusion criteria are: (1) availability of preoperative cervical MRI and CT scans with adequate image quality for VBQ and HU measurements; (2) standing lateral cervical radiographs obtained preoperatively and at least 12 months postoperatively; and (3) absence of systemic disorders affecting bone metabolism.

The exclusion criteria are: (1) history of cervical spine surgery; (2) vertebral deformities due to multi-segment fractures; (3) primary or metastatic spinal tumors; (4) spinal infections; and (5) systemic metabolic bone diseases, including parathyroid dysfunction, thyroid disorders, chronic kidney disease with abnormal mineral metabolism, osteoporosis secondary to rheumatoid arthritis, and long-term glucocorticoid therapy.

### Demographic and clinical data collection

Study collects demographic and clinical information for all enrolled patients, including age, sex, body mass index (BMI), symptom duration before surgery, and baseline functional status assessed using the Japanese Orthopaedic Association (JOA) score and visual analog scale (VAS) score. These variables serve as covariates in the statistical models and are compared between groups to minimize potential confounding effects. Preoperative cervical CT, MRI and x-Ray are obtained within 7 days before surgery.

### Vertebral HU value measurement

**Figure 1:**
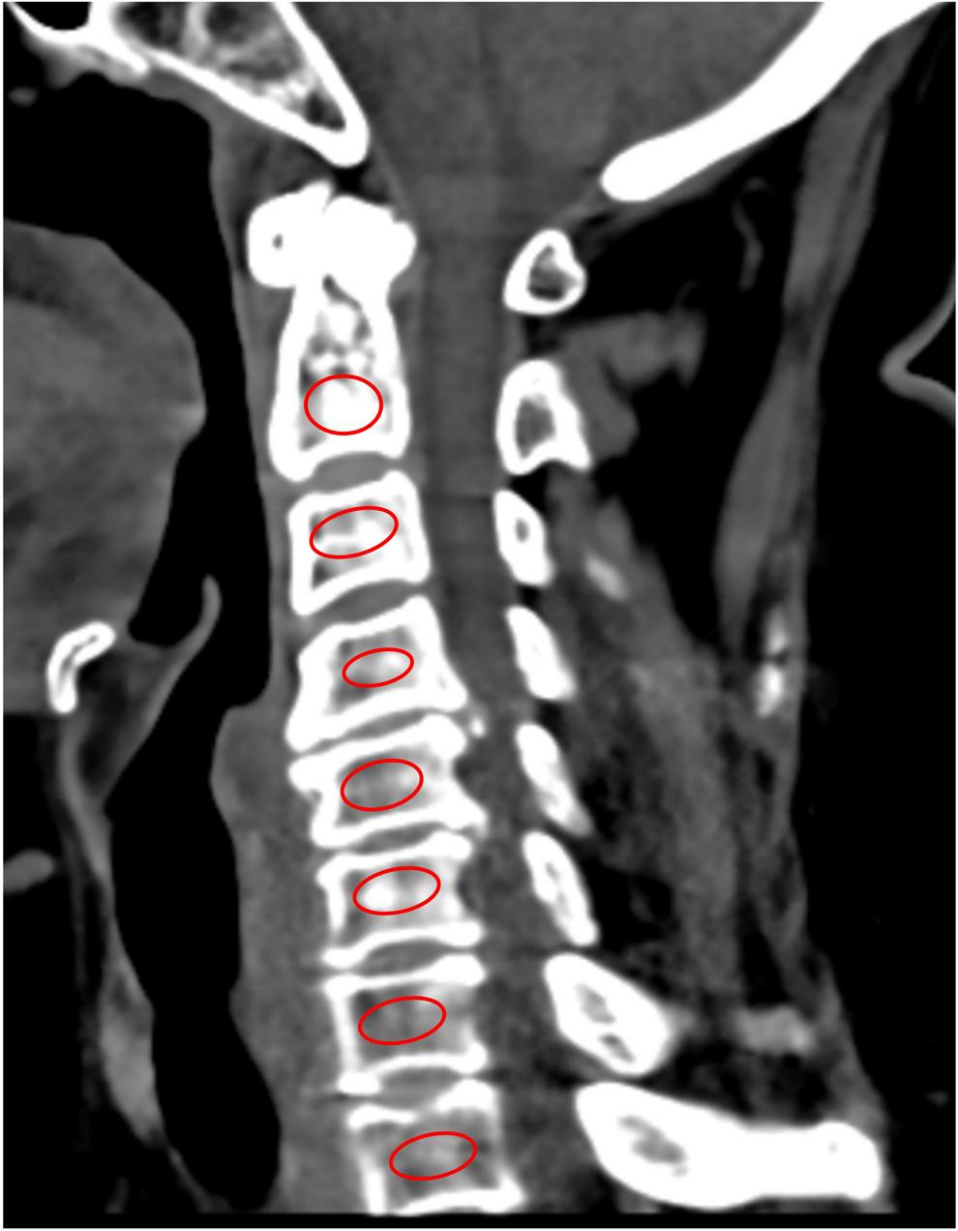
Sagittal computed tomography (CT) image of the cervical spine (C2–T1) showing the placement of elliptical regions of interest (ROIs) for hounsfield unit (HU) measurement. Each vertebral body from C2 through T1 is marked with a red ROI placed centrally within the trabecular bone. The ROIs are positioned to avoid inclusion of the surrounding cortical bone, endplates, and venous plexuses, ensuring that the measured HU values accurately represent trabecular bone density.

The study utilizes preoperative cervical CT plain scan data to assess vertebral HU values (128-slice scanner). On sagittal CT images at the mid-level of each cervical vertebra, a circular or elliptical ROI is placed covering most of the central cancellous bone region of the vertebral body, recording the mean HU value. ROI placement avoids interference from cortical bone, facet joint processes, posterior venous plexus, and other high-density or non-osseous structures as much as possible. The study measures HU values for each vertebra from C2 to T1 and calculates the mean value as the patient's cervical HU value. Two experienced spine surgeons independently measure the images and take the average. Figure 1 demonstrates the HU measurement method.

### Vertebral bone quality (VBQ) measurement

**Figure 2:**
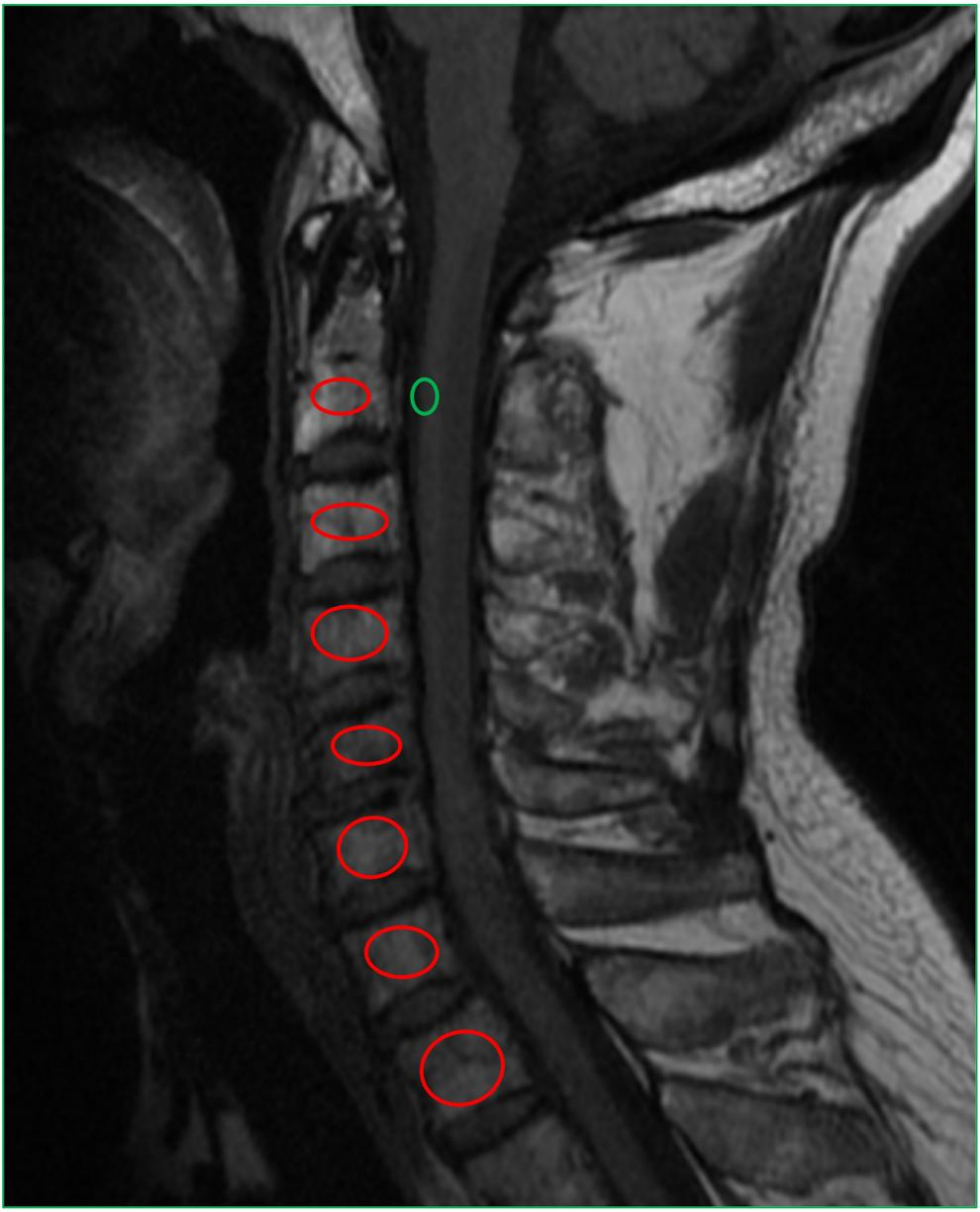
Sagittal T1-weighted magnetic resonance imaging (MRI) scan of the cervical spine (C2–T1) showing ROI placement for vertebral bone quality (VBQ) measurement. Red ROIs are placed within the central trabecular bone of each vertebral body (C2–T1), and an additional green ROI is drawn in the cerebrospinal fluid (CSF) region at the C2 vertebral level as the reference standard. The VBQ value is calculated as the ratio of vertebral body signal intensity to CSF signal intensity, providing a normalized index of bone marrow fat content and vertebral bone quality. Higher VBQ values indicate greater fatty infiltration and lower bone quality.

The study collects preoperative cervical MRI T1-weighted sagittal images acquired on 3.0-T MRI system. A circular ROI is placed in the central cancellous bone region of each assessable cervical vertebra (C2-T1), recording its mean signal intensity. Simultaneously, an ROI is selected within the C2 vertebrae adjacent dural sac to measure cerebrospinal fluid signal intensity as a standard reference. To reduce measurement error, all ROIs maintain similar areas and avoid endplate sclerosis, blood vessels, and other structures. The VBQ value for each vertebra is defined as the ratio of vertebral signal intensity to C2 cerebrospinal fluid signal intensity. The study uses the mean VBQ value from C2 to T1 for each patient in statistical analysis. Two experienced spine surgeons independently measure the images and take the average. Figure 2 demonstrates the VBQ measurement method.

### Imaging measurements and reliability

HU and VBQ are independently and mutually blinded measured by two trained spine surgeons under a standardized protocol. To assess reproducibility, we randomly sample 30 patients from the cohort: HU and VBQ are measured once and repeated after two weeks by the same rater to evaluate intra-observer repeatability, and independently by both raters to evaluate inter-observer agreement. The results are shown in Supplementary Table 1.

### Radiographic parameter collection and grouping criteria

All patients undergo standing lateral cervical radiography preoperatively and during postoperative follow-up. The study measures cervical sagittal parameters, including C2–7 Cobb angle (cervical kyphosis/lordosis angle), C2–7 sagittal vertical axis distance (cSVA, the horizontal distance from the C2 plumb line to the posterosuperior corner of C7), and T1 slope. The study calculates changes in Cobb angle and cSVA at final postoperative follow-up compared with preoperative values. Based on cSVA changes, patients are divided into two groups: the postoperative sagittal balance maintenance group (cSVA increase ≤10 mm) and the balance deterioration group (cSVA increase >10 mm). The primary outcome of this study is postoperative sagittal balance deterioration (yes/no), with the 10 mm threshold referencing previous literature definitions of cervical imbalance.

### Statistical analysis

The study uses SPSS 26.0 software for statistical analysis. All continuous variables undergo normality testing (Kolmogorov–Smirnov test) before analysis. Data following normal distribution are expressed as mean ± standard deviation, with independent samples *t*-tests for group comparisons. Data not following normal distribution are expressed as median (interquartile range), with Mann–Whitney *U* tests for group comparisons. Categorical variables are expressed as frequencies and percentages, with chi-square tests or Fisher's exact tests for group comparisons.

First, patients are divided into three groups (improvement, stable, and deterioration) based on postoperative sagittal balance changes (ΔcSVA), and univariate comparisons are performed for each variable to screen factors showing statistically significant differences among the three groups. Second, variables with significant differences are included in ordinal logistic regression analysis, with the three-category ΔcSVA result as the dependent variable, to explore the independent correlation between each indicator and postoperative sagittal balance changes, thereby determining independent influencing factors.

We tested multiplicative interaction by fitting a logistic regression including standardized HU and VBQ (*z*-scores) and their product term (HU × VBQ). Model fit was compared with the main-effects-only model using a likelihood ratio test (LRT). We additionally evaluated additive interaction as a supplementary analysis by dichotomizing HU and VBQ at the prespecified cutoffs (HU: 308.6; VBQ: 2.92) and estimating risk ratios from a 2 × 2 table with continuity correction and nonparametric bootstrap to derive 95% CIs for relative excess risk due to interaction (RERI), the attributable proportion due to interaction (AP), and the synergy index (*S*). To quantify incremental predictive value, we compared AUCs between single-marker and combined models and generated bootstrap 95% CIs for ΔAUC.

Subsequently, based on significant independent variables from ordinal logistic regression (such as VBQ and HU values), binary logistic regression models are established with postoperative sagittal balance deterioration (ΔcSVA >10 mm) as the dependent variable, calculating predicted probabilities for each patient. The study draws receiver operating characteristic (ROC) curves based on these models, calculates area under the curve (AUC) and 95% confidence intervals, and compares the predictive efficacy of VBQ single indicator, HU single indicator, and their combined model. The maximum Youden index principle determines the optimal cutoff threshold, and the study calculates sensitivity, specificity, positive predictive value, and negative predictive value. Two-tailed tests consider *P* < 0.05 as statistically significant.

## Results

### General data

The study includes 104 patients undergoing cervical laminoplasty, comprising 48 males and 56 females, with a mean age of 58.4 ± 11.6 years and mean follow-up of 22.2 ± 4.8 months. Based on postoperative ΔcSVA changes, patients are divided into three groups: improvement group (ΔcSVA < −10 mm, *n* = 17), stable group (ΔcSVA between −10 and 10 mm, *n* = 50), and deterioration group (ΔcSVA >10 mm, *n* = 37). The three groups show no statistically significant differences in gender, age, duration, BMI, or follow-up time (*P* > 0.05).

Univariate analysis results appear in Table 1. Preoperative mean HU values differ significantly among the three groups (improvement group 345.25 ± 66.11 vs. stable group 298.96 ± 89.90 vs. deterioration group 261.93 ± 39.18, *P* < 0.001), and VBQ values also show significant differences among the three groups (improvement group 2.98 ± 0.19 vs. stable group 2.92 ± 0.17 vs. deterioration group 3.13 ± 0.20, *P* < 0.001). Additionally, preoperative C2–C7 angle and cSVA show statistical differences among the three groups. Postoperative VAS and JOA scores suggest that the deterioration group experiences poorer pain improvement and lighter neurological function recovery (*P* = 0.007, *P* = 0.048).

**Table 1:** Comparison of evaluated outcomes according to the postoperative ΔcSVA.

Outcomes	Improvement group (*n* = 17)	Stability group (*n* = 50)	Deterioration group (*n* = 37)	*P*
HU	345.25 ± 66.11	298.96 ± 89.90	261.93 ± 39.18	<0.001
VBQ	2.98 ± 0.19	2.92 ± 0.17	3.13 ± 0.2	<0.001
Age (years)	56.76 ± 9.12	59.34 ± 11.93	57.95 ± 12.27	0.7
Gender (female/male)	8/9	30/20	18/19	0.477
Duration	16.35 ± 3.24	21.44 ± 2.35	13.51 ± 2.55	0.067
BMI (kg/m^2^)	25.55 ± 3.32	25.09 ± 3.11	26.07 ± 3.82	0.412
Preoperative C2–C7 Angle (°)	18.41 ± 7.91	18.88 ± 9.47	13.53 ± 10.81	0.036
Postoperative C2–C7 Angle (°)	13.41 ± 8.33	12.82 ± 10.60	9.15 ± 10.96	0.202
Preoperative cSVA (cm)	2.65 ± 0.34	2.49 ± 0.60	3.14 ± 0.66	<.001
Postoperative cSVA (cm)	1.53 ± 0.34	3.09 ± 0.63	4.78 ± 0.65	<.001
Preoperative T1 Slope (°)	21.41 ± 5.72	25.47 ± 7.79	25.74 ± 8.11	0.122
Postoperative T1 Slope (°)	31.59 ± 7.09	35.43 ± 7.65	36.04 ± 9.09	0.159
Preoperative VAS Score	3.53 ± 1.84	3.80 ± 1.86	3.35 ± 1.64	0.505
Postoperative VAS Score	1.35 ± 1.73	1.64 ± 1.45	2.51 ± 1.37	0.007
Preoperative JOA Score	11.53 ± 2.27	11.00 ± 2.23	11.08 ± 2.48	0.716
Postoperative JOA Score	16.47 ± 0.51	16.46 ± 0.61	16.11 ± 0.84	0.048
Follow-up Duration (months)	21.41 ± 3.83	22.84 ± 4.78	21.57 ± 5.28	0.38

**Figure 3:**
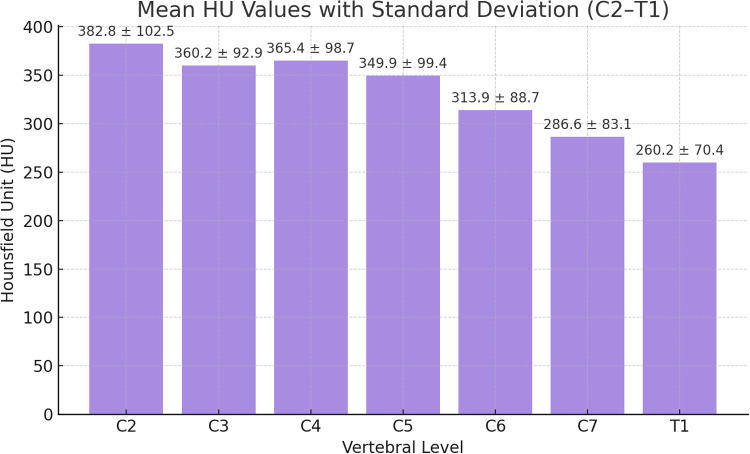
Bar chart showing the mean HU values with standard deviations across cervical vertebral levels (C2–T1). The mean trabecular HU gradually decreases from superior to inferior levels, with the highest density at C2 (382.8 ± 102.5 HU) and the lowest at T1 (260.2 ± 70.4 HU), demonstrating a consistent descending trend in bone density toward the cervicothoracic junction.

Figure 3 displays the distribution pattern of mean HU values from C2 to T1 vertebrae. The cervicothoracic junction region (C7, T1) demonstrates significantly lower HU values than upper cervical vertebrae (C2–C4), showing a gradually declining trend from superior to inferior (mean C2: 382.8 HU decreasing to T1: 260.2 HU).

### Ordinal logistic regression analysis

Indicators showing significant differences in univariate analysis (HU, VBQ, preoperative C2–C7 angle, and preoperative cSVA) are included in the ordinal logistic regression model, with postoperative sagittal balance changes (improvement, stable, deterioration) as the dependent variable. Results show that HU, VBQ, and preoperative cSVA are independent influencing factors for postoperative cSVA deterioration. The regression coefficient for HU value is −0.007 (95% CI: −0.013 to −0.002, *P* = 0.009). The regression coefficient for VBQ is 2.521 (95% CI: 0.432–4.609, *P* = 0.018). The regression coefficient for preoperative cSVA is 0.806 (95% CI: 0.154–1.458, *P* = 0.015), suggesting that a larger preoperative cSVA independently increases the likelihood of postoperative sagittal deterioration. Although the preoperative C2–C7 angle shows a tendency toward association, it does not reach statistical significance (*P* = 0.173).

Table 2 summarizes the ordinal logistic regression results, showing that lower HU, higher VBQ, and greater preoperative cSVA independently predict postoperative sagittal balance deterioration.

**Table 2:** Results of ordinal logistic regression analysis for postoperative deterioration of cSVA.

Variable	Estimate	Wald	95% Confidence Interval	*P*-value
HU	−0.007	6.861	(−0.013, −0.002)	0.009
VBQ	2.521	5.597	(0.432, 4.609)	0.018
Preoperative C2–C7	−0.029	1.999	(−0.069, 0.011)	0.173
Preoperative cSVA	0.806	5.866	(0.154, 1.458)	0.015

### VBQ and HU correlation

**Figure 4:**
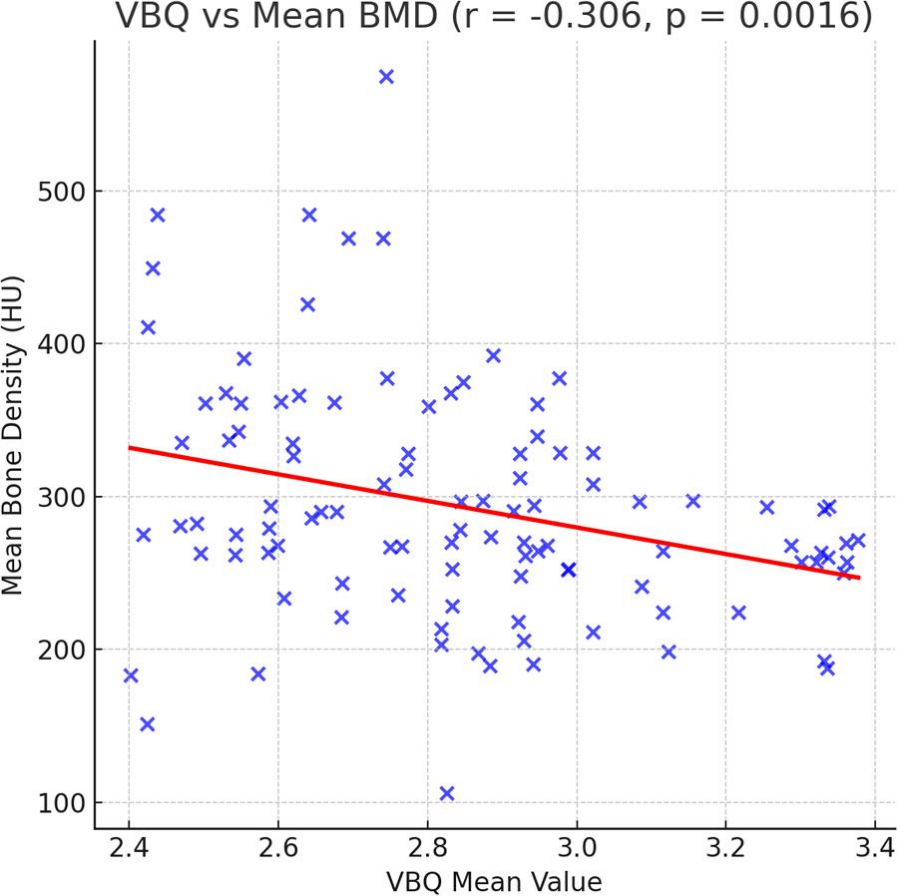
Scatter plot showing the relationship between MRI-derived VBQ values and CT-based mean bone density (HU). A significant negative correlation is observed (*r* = −0.306, *P* = 0.0016), indicating that higher VBQ values (reflecting increased marrow fat content) are associated with lower HU values (indicating reduced trabecular bone density). The red regression line represents the fitted linear model, highlighting the complementary relationship between VBQ and HU in assessing vertebral bone quality.

Figure 4 further demonstrates the linear correlation between preoperative mean VBQ and HU values. The two show significant negative correlation (*r* = −0.306, *P* = 0.0016), indicating that when HU decreases (bone mineral density declines), VBQ values correspondingly increase (bone marrow fat content increases). This trend shows that these two imaging indicators have a complementary relationship in reflecting vertebral bone degeneration, providing theoretical basis for the combined model.

### Predictive performance of VBQ-HU combined model

Based on ordinal regression results, HU and VBQ are included in a binary logistic regression model with “postoperative sagittal balance deterioration (ΔcSVA >10 mm)” as the binary dependent variable, establishing a combined prediction model. Single indicator prediction results show: (1) HU value predicts postoperative sagittal deterioration with AUC of 0.69 (95% CI: 0.591–0.844), optimal cutoff value of 308.6 HU (sensitivity 0.85, specificity 0.65); (2) VBQ value achieves AUC of 0.747 (95% CI: 0.65–0.844), optimal cutoff value of 2.92 (sensitivity 0.865, specificity 0.478).

**Figure 5:**
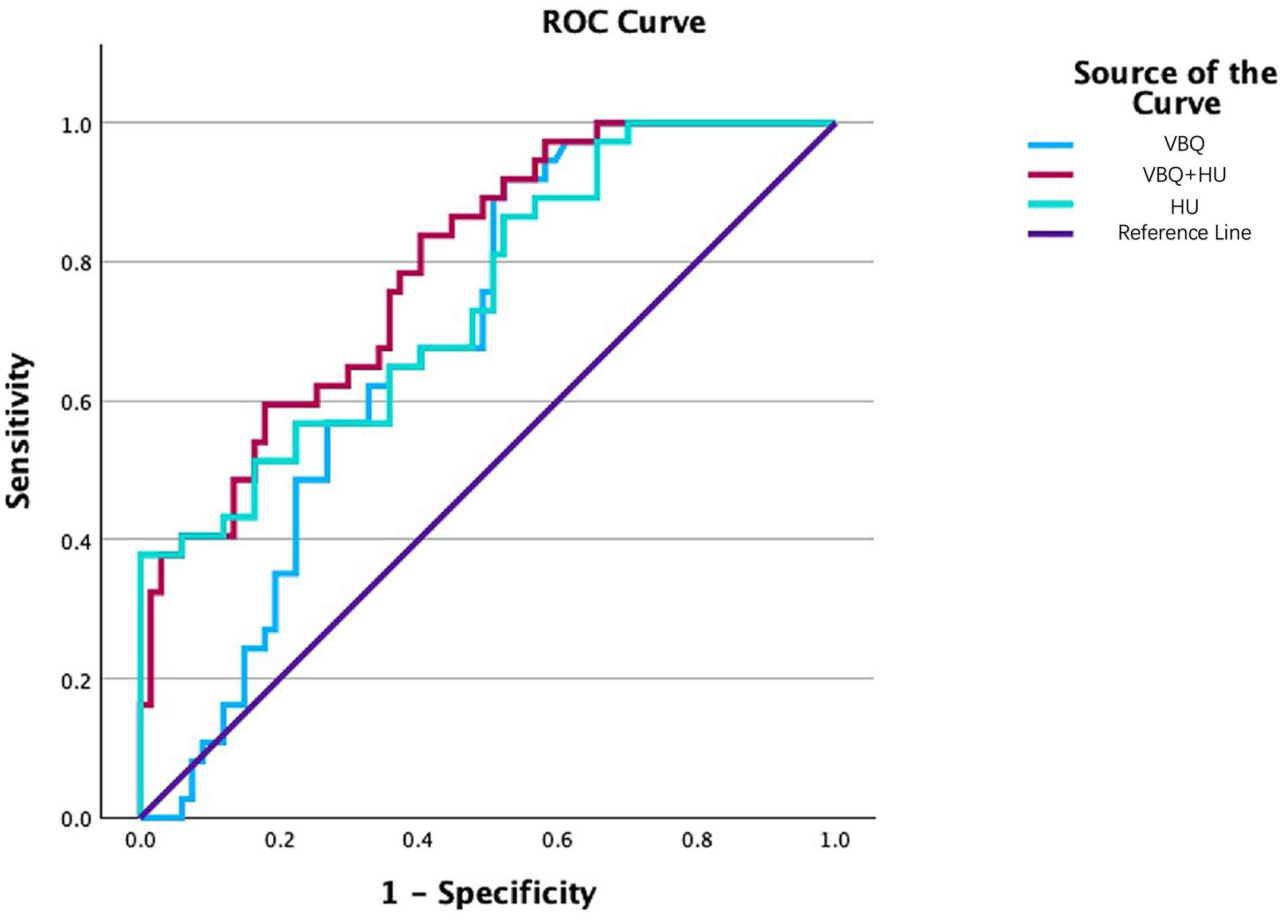
Receiver operating characteristic (ROC) curves comparing the predictive performance of CT-derived hounsfield unit (HU), MRI-derived vertebral bone quality (VBQ), and the combined VBQ + HU model for postoperative sagittal imbalance (ΔcSVA > 10 mm) after cervical laminoplasty. The area under the curve (AUC) is 0.690 for HU, 0.747 for VBQ, and 0.791 for the combined model, indicating improved discriminative ability when both bone quality parameters are integrated. The combined model demonstrates higher sensitivity (0.838) and specificity (0.597) compared with either single-parameter model, supporting the complementary predictive value of HU and VBQ in identifying patients at risk for postoperative sagittal alignment deterioration.

**Figure 6:**
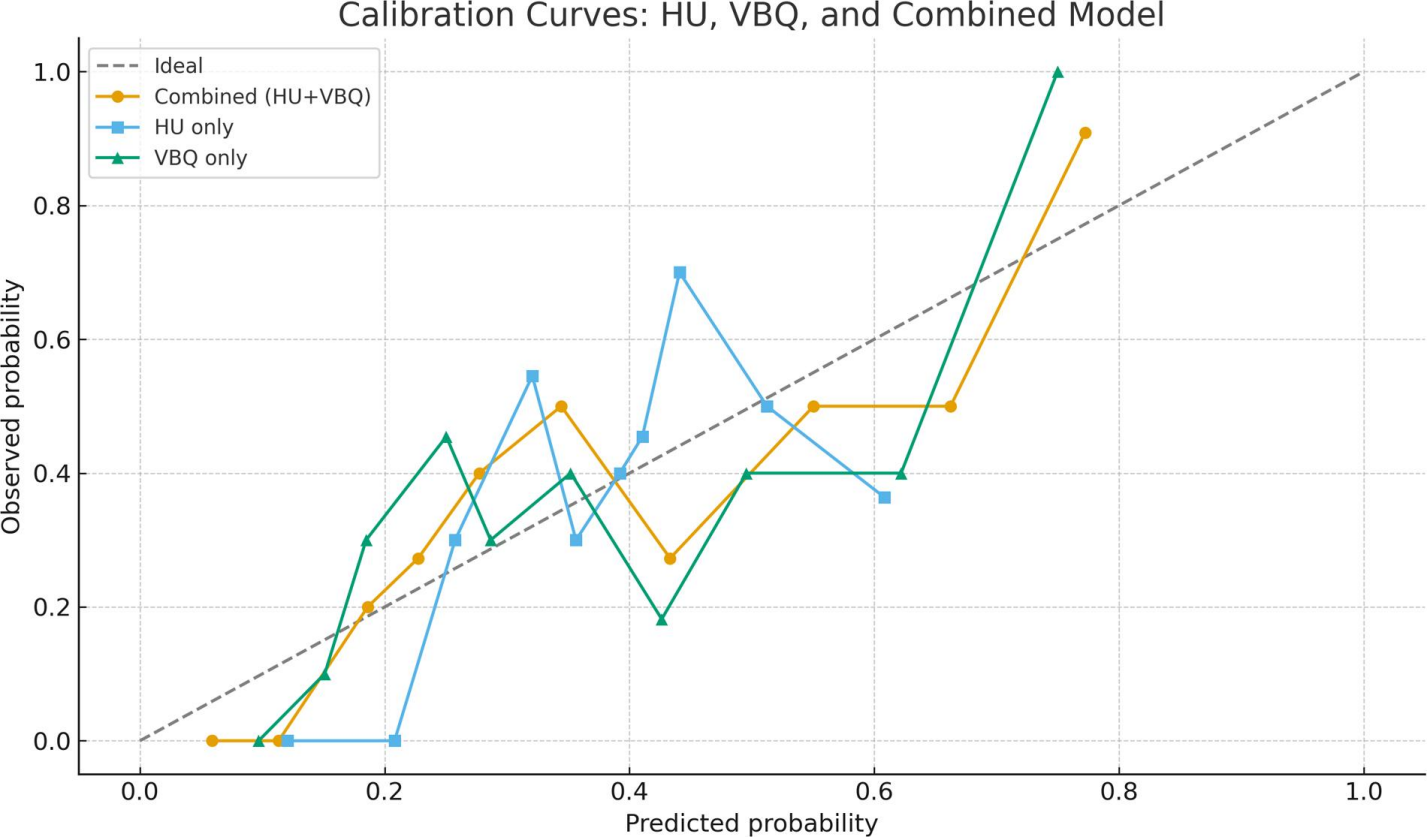
Calibration curves comparing the predictive performance of the HU-only model, the VBQ-only model, and the combined HU + VBQ model for postoperative sagittal balance deterioration.

**Figure 7:**
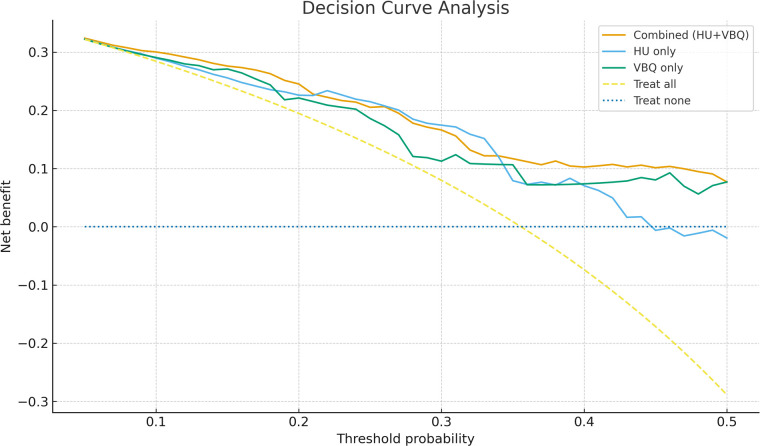
Decision curve analysis evaluating the net clinical benefit of the HU-only model, VBQ-only model, and the combined HU + VBQ model across a range of threshold probabilities.

When the two are combined in modeling, the area under the ROC curve increases to 0.791 (95% CI: 0.706–0.877), with sensitivity 0.838 and specificity 0.597, superior to either single indicator. Table 3 lists ROC parameters for each indicator. The model curves for the three predictive indices appear in Figure 5. Calibration curves are shown in Figure 6. The DCA curves are shown in Figure 7.

**Table 3:** The area under the ROC curve (AUC) and a cutoff value of VBA, HU and combined index.

Parameters	AUC	Cut-off value	Sensitivity	Specificity
VBQ	0.747 (0.65, 0.844)	2.92	0.865	0.478
HU	0.69 (0.591, 0.844)	308.6	0.85	0.65
VBQ-HU	0.791 (0.706, 0.877)		0.838	0.597

To quantify the relative contribution of HU and VBQ within the combined model, we calculated standardized logistic regression coefficients and performed a full dominance analysis. After *z*-score standardization, VBQ demonstrates a stronger standardized effect size than HU within the combined model. VBQ demonstrated a stronger effect size (*β* = 0.985, OR per 1-SD = 2.68), whereas HU showed a smaller standardized effect (*β* = −0.685, OR per 1-SD = 0.50), indicating that VBQ exerts a greater influence on the predicted probability of postoperative sagittal balance deterioration. The results are shown in Table 4.

**Table 4:** Standardized logistic regression analysis of HU and VBQ for predicting postoperative sagittal balance deterioration.

Variable	*β* (Standardized)	SE	Wald	*P*-value	OR per 1-SD	95% CI for OR
HU (per 1 SD decrease)	−0.685	0.245	7.79	0.005	0.50	0.31–0.80
VBQ (per 1 SD increase)	0.985	0.276	12.74	<0.001	2.68	1.58–4.57
Constant	−0.214	0.118	3.29	0.070		

To quantify the relative importance of HU and VBQ in the combined predictive model, we perform a dominance analysis based on likelihood-ratio *χ*^2^ decomposition. The results are summarized in Table 5. The LR *χ*^2^ values for HU alone, VBQ alone, and the full model are 11.05, 21.94, and 28.51, respectively. Shapley value decomposition shows that VBQ contributes 19.70 units of *χ*^2^ (69.1% of total model information), whereas HU contributes 8.81 units (30.9%).

**Table 5:** Dominance analysis based on likelihood-ratio *χ*^2^ decomposition.

Model/metric	HU	VBQ
Single-variable LR *χ*^2^	11.045	21.940
Full model LR *χ*^2^ (HU + VBQ)	28.511	—
Shapley value (Complete dominance)	8.808	19.703
Relative importance (%)	30.9%	69.1%

To evaluate whether HU and VBQ jointly produce synergistic effects on postoperative sagittal balance deterioration, we construct a 2 × 2 exposure matrix (shown in Table 6) using the optimal cutoffs for HU (308.6 HU) and VBQ (2.92), and we calculate interaction measures on both multiplicative and additive scales. The multiplicative interaction term is not statistically significant (*β*_int = −0.066, OR = 0.936, 95% CI: 0.458–1.913; *P* = 0.856). Additive interaction metrics likewise show no significant departure from additivity (RERI = 5.53, 95% CI: −3.65 to 15.36; AP = 0.299, 95% CI: −0.218 to 0.689; *S* = 1.46, 95% CI: 0.81–3.82). The results are summarized in Table 7. These findings indicate that HU and VBQ do not demonstrate statistical synergy; instead, they function as complementary predictors, each providing independent and non-redundant information regarding the risk of postoperative sagittal imbalance.

**Table 6:** Additive interaction 2 × 2 table for postoperative cSVA deterioration. Cells show number of patients with cSVA deterioration (cases) out of total in each HU/VBQ category.

HU/VBQ	VBQ < 2.92 (low)	VBQ ≥ 2.92 (high)
HU > 308.6 (high HU)	1/23 (4.3%)	6/27 (22.2%)
HU ≤ 308.6 (low HU)	4/14 (28.6%)	28/40 (70.0%)

**Table 7:** Multiplicative and additive interaction between HU and VBQ for predicting postoperative sagittal balance deterioration.

Interaction Type	Statistic/Parameter	Estimate	95% CI	*P*-value
Multiplicative interaction	B (interaction term: HU × VBQ)	−0.066		0.856
	OR for interaction	0.936	0.458–1.913	0.856
Additive interaction	RERI	5.53	−3.65 to 15.36	
	AP	0.299	−0.218 to 0.689	
	S	1.46	0.81–3.82	

## Discussion

This study's results show that elevated preoperative vertebral bone quality score (VBQ) and decreased vertebral Hounsfield unit value (HU) both represent independent risk factors for cervical sagittal vertical axis (cSVA) deterioration following cervical laminoplasty. This finding suggests that higher degrees of osteoporosis correlate with poorer postoperative cervical alignment stability. The biological mechanism may lie in the fact that decreased bone mineral density weakens vertebral trabecular bone structure, particularly the loss of horizontal trabeculae, which increases the risk of minor anterior compression fractures and wedge deformities, thereby causing microscopic instability of postoperative vertebral sequences and loss of lordosis (16–18). Simultaneously, during osteoporosis, bone marrow fat infiltration increases, manifesting as signal enhancement on MRI T1-weighted images, which precisely represents the imaging reflection of elevated VBQ index. Increased intraosseous fat content signifies reduced functional trabeculae and decreased skeletal compressive resistance (9, 19). Therefore, whether CT-quantified HU value decrease or MRI-quantified VBQ value increase, both essentially reflect weakened vertebral bone quality, leading to insufficient postoperative cervical structural support and potentially resulting in cervical sagittal deterioration following laminoplasty.

**Figure 8:**
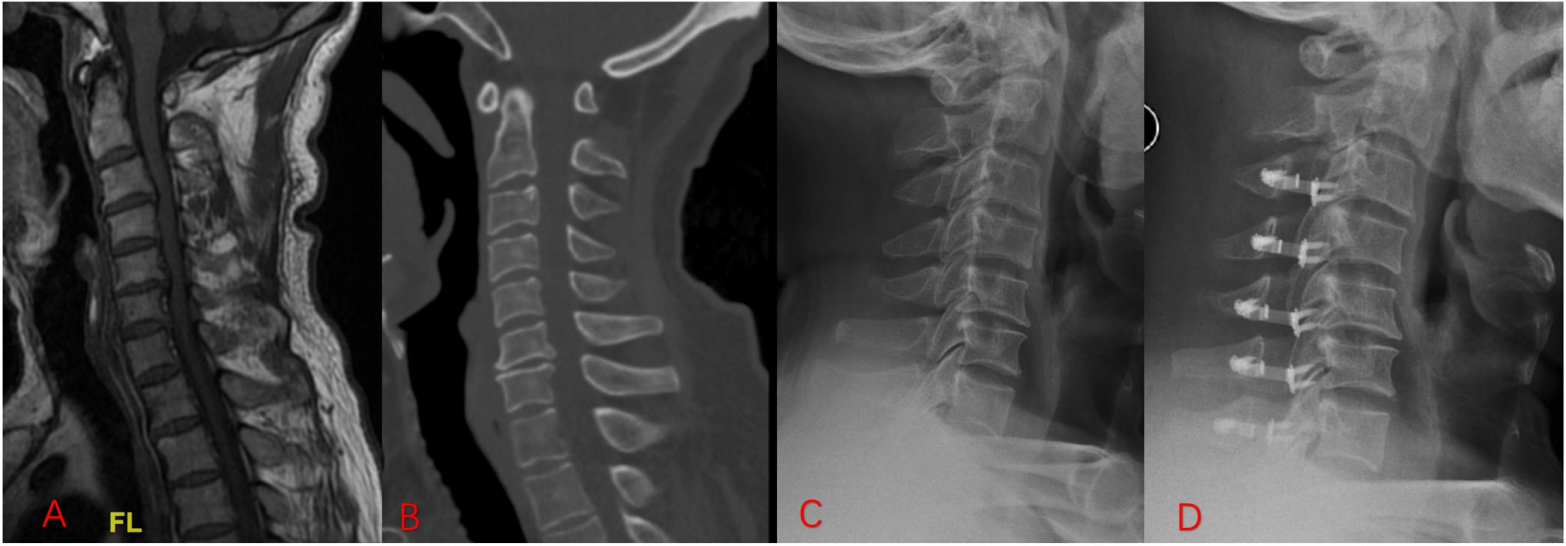
Representative case of postoperative sagittal balance deterioration following cervical laminoplasty. **(A)** Preoperative sagittal T1-weighted MRI showing VBQ 2.72. **(B)** Preoperative sagittal CT image showing reduced cancellous bone density with a mean cervical HU value of 275.43. **(C)** Preoperative standing lateral radiograph showing a cervical sagittal vertical axis (cSVA) of 1.69 cm. **(D)** Postoperative 24-month lateral radiograph showing progression of cSVA to 2.83 cm, defined as sagittal balance deterioration. This patient (male, 68 years old) presented with a VBQ score of 2.72 and HU of 275.43, classifying him within the high-risk group as predicted by the VBQ + HU combined model.

Notably, combined application of VBQ and HU indicators improves prediction accuracy. In this study, using HU or VBQ alone to predict postoperative cSVA deterioration achieves AUC values of 0.69 and 0.747, respectively, whereas combining the two increases model AUC to 0.791, suggesting that combined assessment better identifies high-risk patients. VBQ derives from MRI and reflects bone marrow fat content, while HU comes from CT quantifying bone mineral density. These two represent bone quality information with certain complementarity. The improvement in combined model predictive performance means that preoperatively, simultaneously evaluating both MRI and CT bone quality parameters can more accurately screen patients who may develop postoperative alignment imbalance. For these high-risk patients, clinical practice can consider preoperatively strengthening osteoporosis intervention, adjusting surgical strategies, or enhancing postoperative rehabilitation follow-up to reduce adverse consequences of sagittal imbalance. This finding holds important significance for preoperative risk assessment. Assessment methods combining multimodal imaging indicators promise to improve prediction precision for postoperative sagittal imbalance, thereby improving individualized surgical decision-making and prognosis management. A typical case also supports these findings (Figure 8). The 68-year-old male patient presents with a low HU value (275.43) and an elevated VBQ score (2.72), both indicating impaired vertebral bone quality. Although his preoperative cSVA is normal (1.69 cm), it progresses to 2.83 cm at 24 months, meeting the definition of sagittal imbalance. This example highlights how combined abnormalities in HU and VBQ can preoperatively identify patients at increased risk for postoperative alignment deterioration.

Each imaging-based bone quality parameter provides distinct yet complementary insight into cervical sagittal balance. CT-derived HU values quantitatively reflect the mineral density and structural stiffness of trabecular bone. Lower HU values indicate reduced cancellous bone mineralization and diminished vertebral load-bearing capacity, which may predispose to postoperative segmental settling, wedge deformity, or loss of lordosis under physiological stress. Prior studies have shown that decreased cervical HU is associated with loss of cervical curvature or implant subsidence following laminoplasty and fusion procedures, underscoring its role as a surrogate marker of vertebral mechanical integrity (20, 21).

In contrast, the MRI-derived VBQ score captures the compositional aspect of bone quality by quantifying bone marrow fat infiltration on T1-weighted images. Elevated VBQ values signify increased marrow adiposity and decreased hematopoietic bone volume, representing microarchitectural deterioration not detectable on CT. In the context of cervical alignment, excessive marrow fat weakens trabecular connectivity and impairs the vertebra's ability to resist anterior compressive forces, thereby facilitating postoperative sagittal imbalance (22, 23). Thus, HU predominantly assesses mineralized bone mass, whereas VBQ sensitively reflects fatty degeneration and metabolic quality of the trabecular matrix. Each metric highlights a different biological dimension of vertebral fragility—structural vs. compositional—providing complementary information for predicting postoperative alignment stability.

This study still has some limitations requiring consideration. First, this study represents a single-center retrospective analysis with relatively limited sample size, potentially introducing selection bias, thereby reducing statistical power and the generalizability of conclusions. Future multicenter, large-sample prospective studies are needed to validate our findings and improve evidence levels. Second, this study's follow-up duration is moderate (approximately two years), not yet permitting evaluation of longer-term sagittal changes and clinical impacts. Subsequent studies can extend follow-up to observe long-term effects. Additionally, although VBQ and HU can reflect bone quality, we do not combine them with the gold standard bone mineral density measurement-DEXA, so the evaluation of osteoporosis remains somewhat indirect. Manual ROI delineation may introduce subjective variability in HU and VBQ measurements despite being performed by experienced raters. In future research, semi-automated or algorithm-assisted segmentation pipelines with repeated-measurement reliability assessment are needed to enhance methodological rigor and reproducibility. Finally, our current work focuses on model development and internal validation, and it does not provide a deployable clinical decision tool such as a full logistic equation, risk nomogram, or online calculator. A future phase of this research focuses on translating the model into a user-friendly platform to support real-world clinical decision-making.

Future research directions may include: applying machine learning or artificial intelligence models, integrating bone quality indicators with other patient parameters (such as age, muscle condition, systemic bone metabolism indicators, etc.) to establish more comprehensive prediction models, aiming to further improve prediction accuracy and practicality; and validating the applicability of VBQ and HU prediction models across different ethnic groups and age populations.

## Conclusion

In patients undergoing cervical laminoplasty, higher MRI-derived VBQ and lower CT-derived HU independently predict postoperative cervical sagittal balance deterioration, reflecting complementary aspects of bone degeneration. A combined VBQ + HU model improved discrimination over either metric alone (AUC = 0.791), supporting its use for preoperative risk stratification. Integrating this dual-modality bone assessment into surgical planning may help tailor osteoporosis management and follow-up to reduce alignment loss. Prospective multicenter validation with longer follow-up is warrante.

## Data Availability

The raw data supporting the conclusions of this article will be made available by the authors, without undue reservation.
