# Supplementary material for: Synergistic predictive value of MRI-based vertebral bone quality and CT hounsfield units for postoperative sagittal balance deterioration after cervical laminoplasty
Source: Front Surg. 2025 Dec 18;12:1722804. doi: 10.3389/fsurg.2025.1722804 (PMC12756165; doi:10.3389/fsurg.2025.1722804)
Supplement: Supplementary file 1 [file Table1.docx]

| **Parameter** | **Reliability type** | **ICC value** | **95% CI** |
| --- | --- | --- | --- |
| HU | Inter-observer | **0.88** | **0.79–0.93** |
| HU | Intra-observer | **0.92** | **0.86–0.96** |
| VBQ | Inter-observer | **0.84** | **0.73–0.91** |
| VBQ | Intra-observer | **0.90** | **0.83–0.95** |

Supplementary Table1. Inter- and intra-observer reliability for HU and VBQ measurements
